# Genome-Wide Identification of the R2R3-MYB Gene Family Members in Masson Pine and the Regulation of Secondary Cell Wall Formation and Lignin Biosynthesis by *PmMYB289*

**DOI:** 10.3390/plants15081216

**Published:** 2026-04-16

**Authors:** Qianzi Li, Yidan Song, Sheng Yao, Yuchuan Hu, Laiwang Sun, Kongshu Ji

**Affiliations:** 1 State Key Laboratory of Tree Genetics and Breeding, Nanjing Forestry University, Nanjing 210037, China; qianzili@njfu.edu.cn (Q.L.); syd@njfu.edu.cn (Y.S.); yaosheng0817@njfu.edu.cn (S.Y.); 18751825766@njfu.edu.cn (Y.H.); 17516758856@njfu.edu.cn (L.S.); 2 Co-Innovation Center for Sustainable Forestry in Southern China, Nanjing Forestry University, Nanjing 210037, China

**Keywords:** functional analysis, Masson pine, *PmMYB289*, secondary cell wall, transcription factors

## Abstract

Secondary cell wall (SCW) formation and lignin biosynthesis are critical biological processes that determine wood properties. Masson pine (*Pinus massoniana* Lamb) is a fast-growing conifer species with significant economic value for the pulp and paper industry. While R2R3-MYB transcription factors are known as master regulators of SCW biosynthesis, the specific R2R3-MYB members regulating lignin formation in Masson pine remain largely uncharacterized. In this study, we identified 317 R2R3-MYB genes in the Masson pine genome. Phylogenetic analysis revealed that *PmMYB289*, a member of the P20 subgroup, is highly homologous to the *Arabidopsis* SCW regulators *AtMYB52* and *AtMYB54*. Expression profiling demonstrated that *PmMYB289* is predominantly expressed in highly lignified old stems. Transcriptional activation assays confirmed that *PmMYB289* lacks autoactivation activity. Subcellular localization analysis revealed that *PmMYB289* was localized to the nucleus. Ectopic overexpression of *PmMYB289* in tobacco (*Nicotiana benthamiana*) resulted in dwarfed plant growth, reduced stem diameter, and curled leaves. Molecular analysis of these transgenic lines showed a significant downregulation of most key SCW biosynthetic genes, with the exception of *NbPAL1*. These findings indicate that PmMYB289 acts as a crucial transcriptional repressor in SCW biosynthesis, providing valuable genetic resources for the molecular breeding of superior Masson pine varieties.

## 1. Introduction

The MYB transcription factor family ranks among the largest gene families in plants, playing diverse roles in development, defense, and primary and secondary metabolism. The highly conserved N-terminal DNA-binding domain of these proteins typically consists of imperfect amino acid repeats (R) [1], with each repeat containing three regularly spaced tryptophan residues that form a hydrophobic core essential for specific DNA recognition [2]. Among the subclassifications, the R2R3-MYB subfamily is the most abundant in plants [3,4,5]. During wood formation, which involves vascular cambium differentiation, secondary cell wall (SCW) deposition, and programmed cell death [6], the changes in cell wall composition are strictly regulated [7]. Within this process, R2R3-MYB transcription factors function as critical second-level master switches within a hierarchical regulatory network [8,9,10,11,12], directly governing the ectopic deposition of lignin, cellulose, and hemicellulose [9].

In this hierarchical network, specific R2R3-MYB members have been characterized as central regulators. For instance, in *Arabidopsis thaliana*, the genes *AtMYB46* and *AtMYB83* encode key master switches [13,14] that directly activate downstream transcription factors and structural genes to promote SCW thickening in vessels and fibers [15,16]. Homologous genes in other woody species also exhibit similar functions in SCW regulation [17,18,19,20]. However, the transcriptional regulatory networks can diverge significantly among species. For example, *AtMYB46* directly activates the expression of genes encoding enzymes and structural proteins involved in secondary cell wall biosynthesis, thereby actively promoting the formation of secondary cell walls in fibers and ducts [8]. Among the 29 direct target genes of *Populus trichocarpa PtrMYB021*, the cellulose synthase gene *PtrCESA* and the phenol oxidase gene *PtrLAC* are both directly activated by *PtrMYB021*, and they are functionally essential for the biosynthesis and assembly of secondary cell walls in poplar cells [8]. Furthermore, transcription factors can play distinct or even opposite roles depending on the plant species. For example, the homologous gene *PtrMYB189/090/158/031/161/175* in *P*. *trichocarpa* plays a negative regulatory role in the synthesis of secondary cell walls [21,22], while in *Arabidopsis thaliana*, its homologous genes *AtMYB52* and *AtMYB54* exert a positive regulatory effect [16], which means the regulation by SCW is species-specific. This evolutionary divergence underscores the necessity of characterizing these regulatory networks directly within target tree species rather than relying solely on model organisms.

Masson pine is a fast-growing conifer and a vital timber resource for the pulp and paper industry due to its excellent wood properties and high cellulose content [23,24]. Despite its tremendous economic importance and the recent publication of its large genome [25], the genetic mechanisms and the specific R2R3-MYB transcription factors regulating SCW biosynthesis in Masson pine remain largely uncharacterized, mainly hindered by its resistance to genetic transformation [24]. We hypothesize that specific R2R3-MYB members in Masson pine function as core regulators of lignin and cellulose deposition, utilizing mechanisms that may be distinct from their angiosperm orthologs. Therefore, this study aims to perform a genome-wide identification of the R2R3-MYB gene family in Masson pine and explicitly characterize the functional role of *PmMYB289*. By evaluating its expression patterns and ectopically overexpressing *PmMYB289* in transgenic tobacco, we seek to elucidate its regulatory function in SCW and lignin biosynthesis, providing a solid theoretical foundation for the genetic improvement of pulpwood varieties in coniferous species.

## 2. Results

### 2.1. Identification of the R2R3-MYB Subfamily in Masson Pine and the Construction of the Phylogenetic Tree

**Figure 1:**
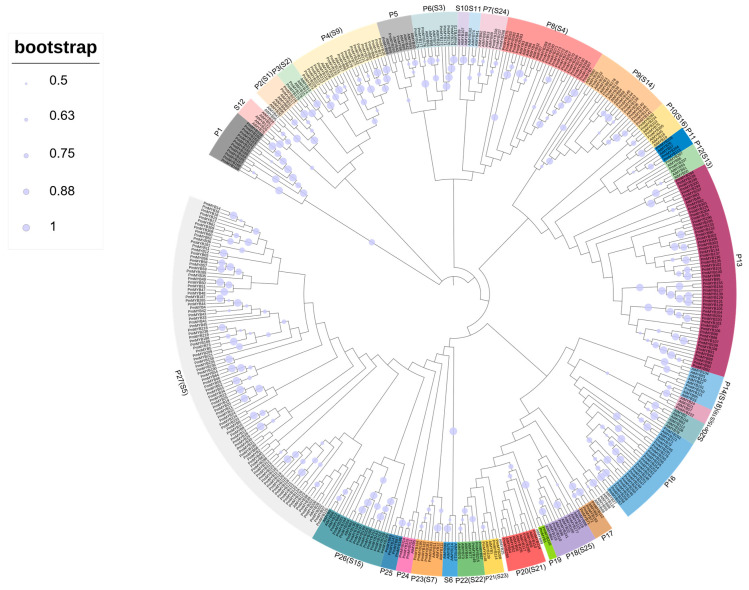
Phylogenetic tree construction of Masson pine and *Arabidopsis thaliana* R2R3-MYB family members. Different colors represent different subgroups. P stands for Masson pine; S stands for *Arabidopsis thaliana*. The bootstrap values were represented by purple solid circles in proportion to size, with the largest circle corresponding to 1.0 (100%), followed by 0.88 (88%), 0.75 (75%), 0.63 (63%), and the smallest circle corresponding to 0.5 (50%).

The protein sequences of the R2R3-MYB gene family in *Arabidopsis* were downloaded from the PlantTFBD database. By comparing the protein sequences with those of the genome protein of Masson pine, 663 sequences were obtained. Sequences without conserved domains and those with incompletely conserved domains were removed, and redundant sequences were eliminated using MEGA X. Overall, 317 members of the PmMYB gene family in Masson pine were determined (Table S2). To explore the phylogenetic relationship, a phylogenetic tree was constructed based on the sequence alignment of 317 PmMYB proteins and 125 AtMYB proteins (Figure 1). The reliability of nodes was evaluated using the neighbor-joining method and 1000 confidence repetitions. Nodes with a bootstrap value less than 50% were not displayed. Most of the core nodes in the phylogenetic tree showed high bootstrap support values, confirming the reliability of the clustering results. Based on the chromosome localization results, the 317 PmMYB members were named *PmMYB1* to *PmMYB317* (Table S1). S1 to S25 indicated the homologous subfamilies of AtR2R3-MYB genes, used for annotating the evolutionary homology between PmMYB and AtMYB subfamilies, where proteins of *Arabidopsis* without color marking were not assigned to known *Arabidopsis* subfamilies [26]. In the phylogenetic tree, the clustering pattern of the R2R3-MYB subfamily of Masson pine showed a certain similarity to that of *Arabidopsis*. Additionally, combining the protein sequence similarity and the high bootstrap support degree of the clustering results, the PmMYB genes were further divided into 27 evolutionary branches, labeled as P1 to P27 subgroups. Among the 27 PmMYB subgroups, P27 contained the largest number of family members, with 99, while P15 had the fewest members, with only one.

### 2.2. Chromosome Localization of the R2R3-MYB Subfamily of Masson Pine

**Figure 2:**
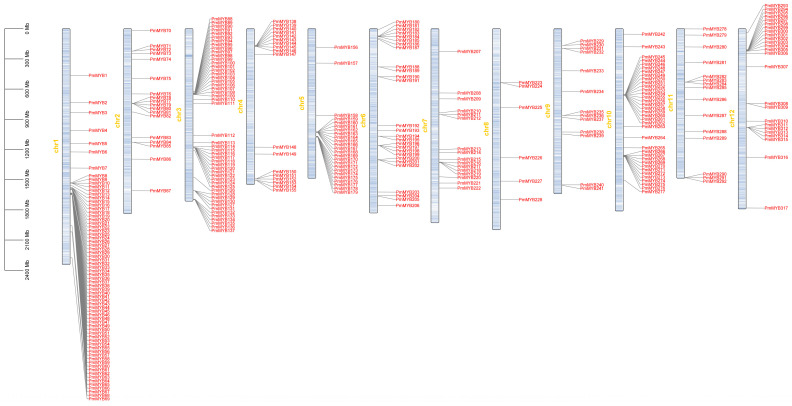
Chromosomal distribution map of R2R3-MYB subfamily transcription factor family in Masson pine across 12 chromosomes. Chr1-chr12 represent the 12 chromosomes of Masson pine. The vertical axis on the left is the physical length scale of chromosomes, with the unit of megabase (Mb). Naming PmMYB1-317 based on the position of the gene on the chromosome.

Members of the Masson pine R2R3-MYB subfamily are mapped on chromosomes 1 to 12. The distribution of genes on each chromosome is relatively uneven, with more genes on chromosomes 1, 3, 5, and 10, and an uneven distribution of gene positions on each chromosome. Chromosome 1 is the most numerous, with 69 members of the R2R3-MYB subfamily located there; there are the fewest on chromosome 8, with six R2R3-MYB subfamily members. In addition, genes form gene clusters on all chromosomes. According to the analysis of the evolutionary tree, members of the gene clusters have higher homology and are presumed to have relatively conserved functions among them (Figure 2).

### 2.3. Analysis of the Gene Structure and Conserved Motifs of the R2R3-MYB Subfamily of Masson Pine

**Figure 3:**
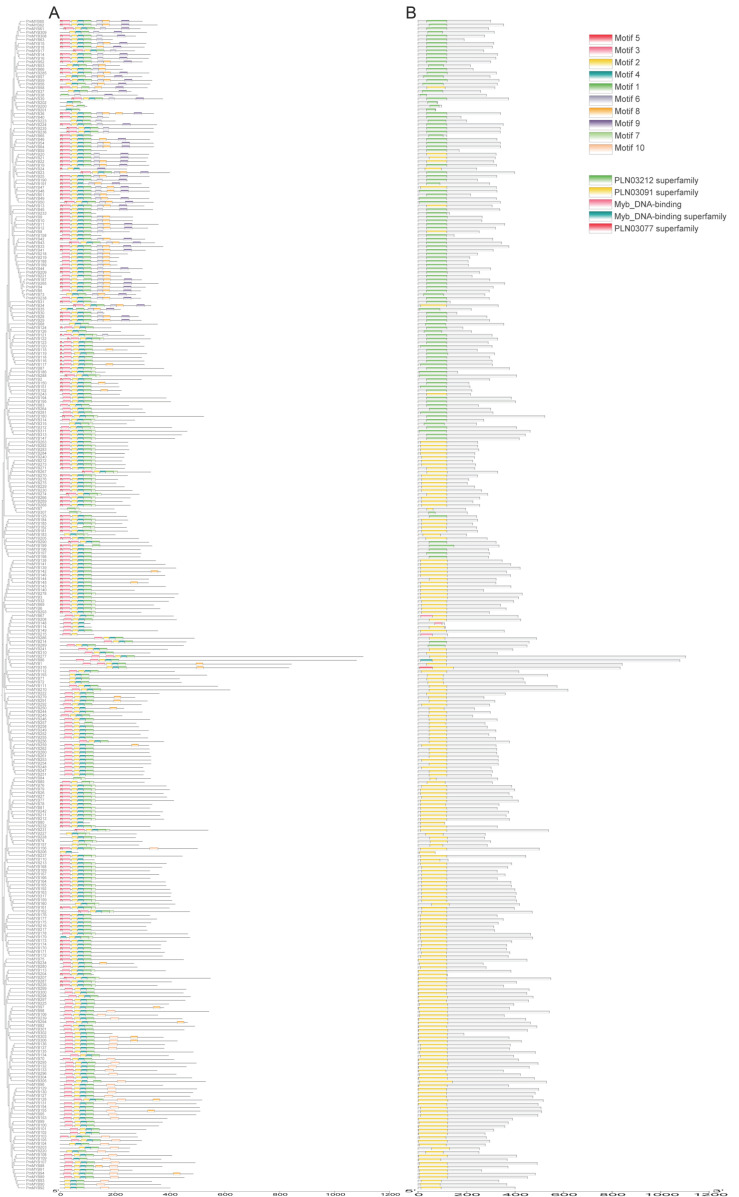
Protein conservative motifs and gene structure of R2R3-MYB subfamily in Masson pine. (**A**) Conserved MOTIF; (**B**) gene structure and conserved domain.

Conservative motif analysis was conducted on the R2R3-MYB subfamily of Masson pine (Figure 3). The results showed that 10 different motifs were identified in 317 R2R3-MYB proteins, among which Motif5, Motif3, Motif2, Motif4 and Motif1 were highly conserved motifs, and all members contained either Motif4 or Motif1 or both. All genes contain the conserved R2R3-MYB domain, resulting in functional similarities among the members. In this study, *PmMYB289* is located in the P20 subgroup, and the gene structures within the same subgroup are similar (Figures S1 and S2). The research indicates that the members of the R2R3-MYB subfamily of Masson pine have specific or identical patterns, which leads to specific or similar functions among them.

### 2.4. PmMYB289 Is Mainly Expressed in Old Stems

**Figure 4:**
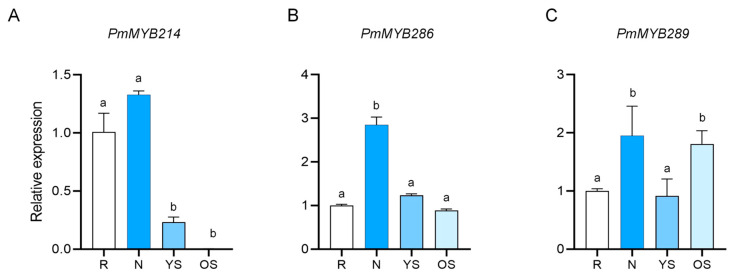
Analysis of the relative expression of (**A**) *PmMYB214*, (**B**) *PmMYB286*, and (**C**) *PmMYB289* in different tissues. R: root; N: needles; YS: semi-lignified stem; OS: lignified stem. Error bars indicate the mean ± standard deviation (SD) based on three biological replicates. Significant differences at *p* < 0.05 (one-way ANOVA) are marked by distinct lowercase letters.

There are three homologous genes to *AtMYB52* and *AtMYB54* related to *Arabidopsis* lignin [16] and secondary wall metabolism in the MYB genome of Masson pine, namely *PmMYB214*, *PmMYB286* and *PmMYB289*. We used RT-qPCR technology to examine the expression levels of these three genes in different tissues. Results showed that all three genes we chose were expressed in the tissues examined, but they exhibited different expression patterns. Notably, *PmMYB286* had the highest relative expression level in needles, while *PmMYB289* maintained high expression levels in needles and lignified stems (old stems) (Figure 4). In fact, the expression level of *PmMYB289* in old stems was about twice that in roots (Figure 4). Since old stems are highly lignified tissues, this specific expression profile strongly indicates that *PmMYB289* plays a critical role in lignin biosynthesis during secondary cell wall formation.

### 2.5. Analysis of Transcriptional Activation Activity of PmMYB289

**Figure 5:**
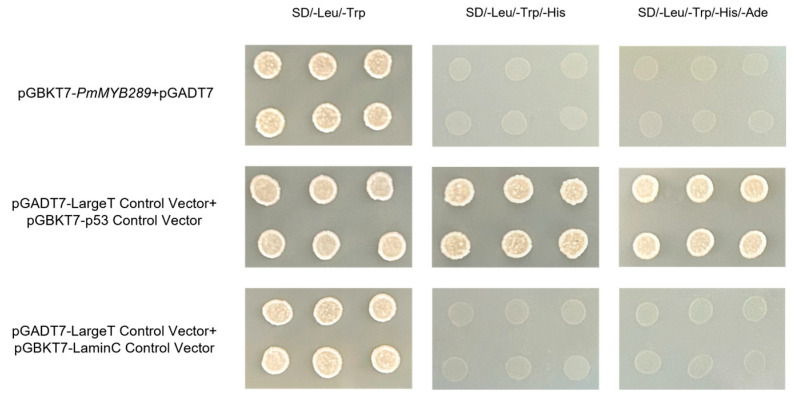
Transcriptional activation analysis of *PmMYB289* in yeast. Yeast cells co-transformed with the indicated plasmids were spotted onto double-dropout (SD/-Leu/-Trp), triple-dropout (SD/-Leu/-Trp/-His), and quadruple-dropout (SD/-Leu/-Trp/-His/-Ade) media. The pGBKT7-*PmMYB289* + pGADT7 construct was the experimental group. “pGADT7-LargeT + pGBKT7-p53” was selected as the positive control, and “pGADT7-LargeT + pGBKT7-LaminC” was selected as the negative control.

To verify whether *PmMYB289* has transcriptional activation, vectors of pGBKT7-PmMYB289 were constructed and co-transformed with pGADT7 into yeast strain AH109. The results showed that yeast cells co-transformed with the recombinant plasmid pGBKT7-*PmMYB289* + pGADT7, as well as the negative control, could only grow on the double-dropout medium (SD/-Leu/-Trp) and failed to grow on the quadruple-dropout medium (SD/-Leu/-Trp/-His/-Ade). In contrast, the positive control grew normally on both media. The above experiments demonstrated that *PmMYB289* was not self-activating (Figure 5).

### 2.6. Subcellular Localization of PmMYB289

**Figure 6:**
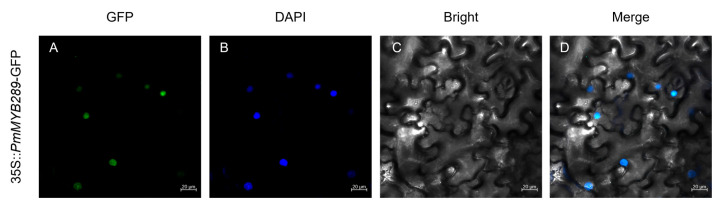
The subcellular localization of *PmMYB289* in tobacco leaves. (**A**) GFP fluorescence, (**B**) DAPI staining of the nucleus, (**C**) bright field, and (**D**) merged image. Bar = 20 μm.

To investigate the localization of the *PmMYB289*, the 35S::*PmMYB289*-GFP fusion construct was used, and it was transfected into tobacco leaves through *Agrobacterium* infection. Fluorescence signals from the *PmMYB289*-GFP fusion protein were exclusively detected in the nucleus, demonstrating that PmMYB289 is a nuclear-localized protein that functions within the nucleus, which was consistent with its predicted role as a transcription factor (Figure 6).

### 2.7. Overexpression of PmMYB289 Affects the Growth of Transgenic Tobacco Plants and the Expression of Genes Related to Secondary Wall Synthesis

**Figure 7:**
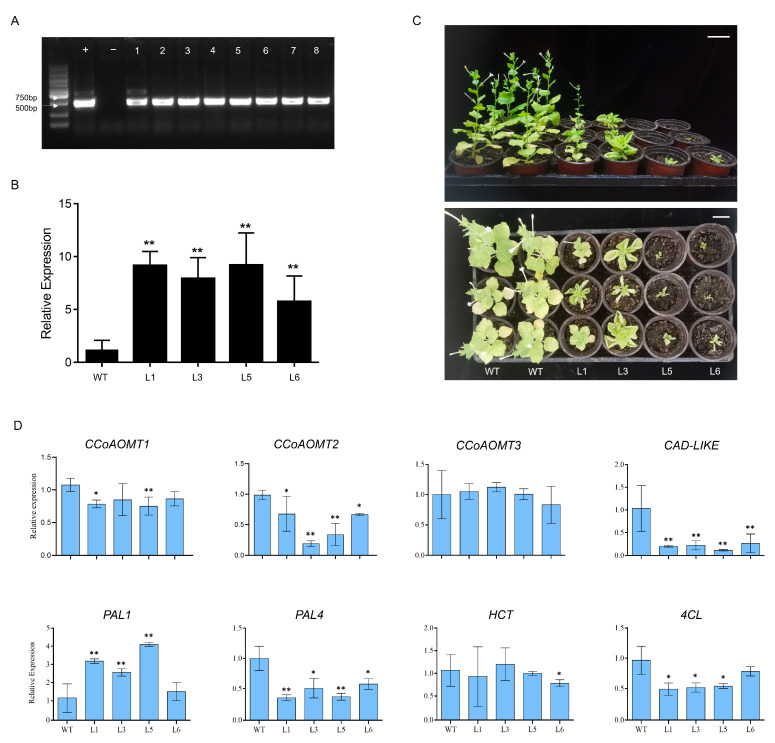
Analysis of relative expression levels and phenotypes of *PmMYB289* overexpression (OE) plants. (**A**) The gDNA verification of overexpressed tobacco, with “+” indicating the use of Masson pine as the positive control, “−” indicating the use of WT tobacco as the negative control, and 1–8 representing different overexpression lines. (**B**) Relative expression levels in wild-type (WT) tobacco and transgenic lines (L1, L3, L5, and L6). (**C**) Phenotypes of WT and transgenic plants: Front view and top view. Bar = 5 cm. (**D**) Relative expression analysis of genes involved in secondary cell wall biosynthesis in *PmMYB289*-overexpressing plants. Error bars indicate the mean ± SD of three biological replicates. Asterisks represent significant differences compared with the wild type based on one-way ANOVA (* *p* < 0.05, ** *p* < 0.01).

The gDNA verification showed that *PmMYB289* was expressed in both the positive control of Masson pine and the overexpressed tobacco, while no expression of *PmMYB289* was observed in the negative control of WT tobacco (Figure 7A). The expression levels of the plants overexpressing *PmMYB289* were detected, and it was found that L1, L3, L5 and L6 had high expression levels (Figure 7B). To investigate whether the overexpression of *PmMYB289* would affect the growth of plants, we observed the individual growth conditions of four transgenic lines and the unmodified control plants. After planting these plants in nutrient soil for one month, the plants overexpressing *PmMYB289* showed significant differences in growth phenotypes compared to the wild-type plants (Figure 7C). Compared to the wild-type plants, the lateral growth of the transgenic plants was significantly inhibited (Figure 7C). At the same time, a considerable proportion of the overexpressing plants exhibited growth arrest, vertical growth was inhibited, and there was no obvious trunk development (Figure 7C). Additionally, the leaf margins of the transgenic plants also showed upward curling (Figure 7C). The results indicate that the overexpression of *PmMYB289* inhibited the normal growth of tobacco.

In the *PmMYB289*-overexpressing tobacco lines, the expression levels of most key enzyme genes involved in secondary wall synthesis were down-regulated to varying degrees (Figure 7D). However, there were notable exceptions: *NbPAL1* was significantly up-regulated, and *NbCCoAOMT3* showed no significant changes across the transgenic lines. Additionally, *NbHCT* expression remained largely unaltered in most lines, with only a slight but significant decrease observed specifically in line L6 (Figure 7D). The results suggest that *PmMYB289* is highly likely to inhibit the biosynthesis of the secondary wall in overexpressed tobacco by suppressing the expression of enzymes related to secondary wall synthesis in transgenic tobacco, but the specific mechanism remains to be explored in the future.

## 3. Discussion

R2R3-MYB transcription factors are widely recognized as pivotal regulators involved in plant abiotic stress responses [27]. Previous studies have documented that most plant species harbor 70 to 200 R2R3-MYB family members [28]. Our study identified 317 R2R3-MYB transcription factors from the Masson pine genome, more than 244 proteins in soybean [29], 192 in black poplar [30], 167 in flax [31], 96 in plum [32], and 94 in pineapple [33]. These results suggest that there are significant variations in the quantity of R2R3-MYB genes among different species. In addition, some MYB proteins lack a complete R2R3 domain architecture, which matches Song’s findings [34].

Phylogenetic relationship-based identification of homologous genes in plants facilitates the prediction of gene functions [35]. To further explore the biological roles of R2R3-MYB transcription factors in Masson pine, we constructed a phylogenetic tree by aligning PmMYB proteins with R2R3-MYB proteins derived from *Arabidopsis*. The majority of PmMYB proteins were clustered into functional groups of *Arabidopsis*, which were categorized into 24 subfamilies, S1–S24. For instance, members belonging to subfamily S1 are involved in the response to both biotic and abiotic stresses [26], members of subgroup S5 participate in the anthocyanin biosynthetic pathway [36], members of subgroup S9 take part in the regulation of trichome formation [37], and members of subgroup S21 play a key role in lignin biosynthesis [38]. Similar to many processes of cell development and differentiation, wood formation involves the interaction of multiple levels of transcription factors with target genes [8,39,40], among which the R2R3-MYB subgroup is an important member. Our phylogenetic analysis reveals that the P20 subgroup in Masson pine clusters closely with the S21 subfamily of *Arabidopsis*. Accordingly, *PmMYB289*, as a core member of the P27 subgroup, exhibits high homology to the *Arabidopsis* genes *AtMYB52* and *AtMYB54*, as well as the *P. trichocarpa* genes *PtrMYB189/090/158/031/161/175* [16,21,22]. Furthermore, our subcellular localization assay confirmed that *PmMYB289* exclusively resides in the nucleus (Figure 6), which is an essential prerequisite for its function as a transcription factor.

Due to the long growth cycle and complex genetic background of coniferous trees, we inferred the function of *PmMYB289* through heterologous overexpression in tobacco plants, a strategy successfully employed in previous studies [16,21]. In our study, the transgenic tobacco overexpressing *PmMYB289* exhibited significantly inhibited growth and leaf curling, which is consistent with the typical phenotype of plants with low lignin content [21,22,41,42]. Interestingly, the homologous genes *PtrMYB189/090/158/031/161/175* in *P. trichocarpa* also negatively regulate the synthesis of secondary cell walls [21,22]. This is opposite to the positive regulatory role of their homologous genes *AtMYB52* and *AtMYB54* in *Arabidopsis* [16]. The relative expression of key enzyme genes in the secondary wall synthesis pathway was further examined, and it was found that, except for *NbPAL1*, which was upregulated, *NbCCoAOMT3* and *NbHCT* showed no significant changes in most lines, and the expression levels of the other genes were significantly suppressed.

Given that the PmMYB289 protein lacks transcriptional autoactivation activity in yeast, yet its overexpression profoundly downregulates the majority of SCW-related structural genes in transgenic tobacco, we postulate that it functions intrinsically as a transcriptional repressor. On the one hand, PmMYB289 may operate as a passive competitive inhibitor. By exhibiting a high binding affinity for specific MYB-recognition elements, such as SMREs, within the promoters of target genes like *NbCCoAOMT1/2* and *NbPAL4*, it could physically obstruct endogenous transcriptional activators from accessing these functional cis-elements. On the other hand, it might act as an active repressor by recruiting highly conserved co-repressor complexes to the chromatin, thereby actively silencing downstream transcription. Subsequent investigations, including the identification of potential repressor domains, for example, the EAR motif, within its sequence or in vivo protein–protein interaction screenings, are indispensable for fully deciphering its precise repressive cascade.

**Figure 8:**
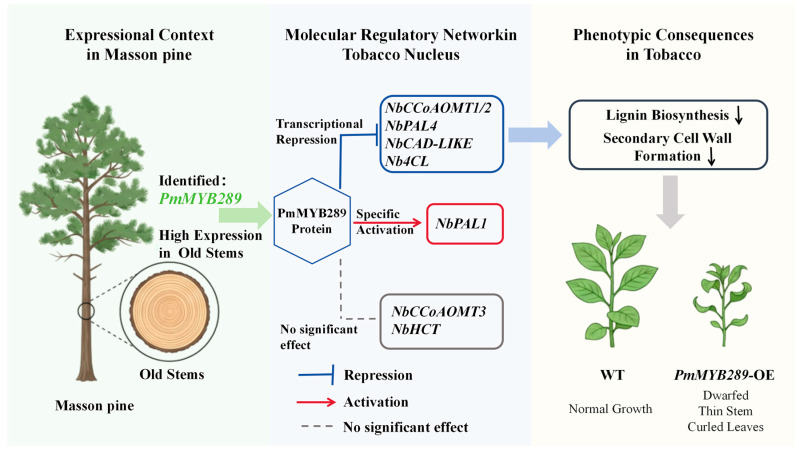
A proposed model for the regulatory mechanism of *PmMYB289* in secondary cell wall and lignin biosynthesis. Based on the identification in Masson pine, *PmMYB289* is highly expressed in old stems. In the transgenic tobacco system, the PmMYB289 protein acts as a transcriptional repressor in the nucleus, significantly downregulating structural genes involved in lignin synthesis, such as *NbCCoAOMT1/2*, *NbPAL4*, *Nb4CL*, and *NbCAD-LIKE*, while showing specific activation of *NbPAL1*. The resulting decrease in lignin and SCW formation leads to phenotypic changes such as dwarfing, thinner stems, and leaf curling. T-shaped bars indicate repression, regular arrows indicate activation, and dashed lines indicate no significant effect.

To synthesize our findings, we propose a comprehensive regulatory model for the role of *PmMYB289* in secondary cell wall biosynthesis (Figure 8). *PmMYB289* is highly expressed in lignified old stems. In the transgenic tobacco system, the PmMYB289 protein acts as a transcriptional repressor in the nucleus, significantly downregulating the core lignin biosynthetic network, which ultimately leads to obstructed SCW formation and macroscopic phenotypic defects.

Although this article provides strong molecular and phenotypic evidence, there are still some limitations. The functional characterization of this study was accomplished through heterologous overexpression in tobacco. Due to the differences in the evolutionary process between gymnosperms and herbaceous dicotyledonous plants, there may be species-specific regulatory differences. Moreover, although the macroscopic phenotype and transcriptome changes strongly indicate that the biosynthesis of the lateral sclerified wall is blocked, future studies need to measure the cell wall thickness and combine with precise histochemical methods to quantitatively analyze the lignin content in the homologous transgenic system, in order to fully elucidate the specific impact of *PmMYB289* on cell biology. In Masson pine, identifying PmMYB289 as a major regulator involved in wood formation is of great significance for the basic research of coniferous trees and also provides possibilities for the potential application of tree biotechnology. Further exploration of the intricate transcriptional regulatory networks in coniferous species is essential to better elucidate the degree of conservation between the transcriptional networks of gymnosperms and angiosperms.

## 4. Materials and Methods

### 4.1. Identification and Categorization of the R2R3-MYB Gene in Masson Pine

The protein sequences of the R2R3-MYB gene family in *Arabidopsis* were obtained from the Plant TFBD database, while the genomic data of Masson pine was from the published genome database [25]. The sequences of *Arabidopsis* were compared with the genomic proteins of Masson pine using the BLAST in TBtools-II. The HMMER (Hidden Markov Model) corresponding to the MYB binding domain (PF00249) was retrieved from the Pfam database (http://pfam.xfam.org/, accessed on 17 January 2026) with an E-value requirement of less than 10^−5^ [43]. This model was used to identify all MYB family transcription factors in Masson pine. The conserved domains of R2R3-MYB proteins in candidate proteins were identified using the online tools NCBI CD Search and Pfam, and sequences without conserved domains or with incompletely conserved domains were removed. Redundant sequences were removed using MEGA X to obtain the final sequences (Table S2).

### 4.2. Phylogenetic Analysis of Masson Pine R2R3-MYB Protein

Sequence alignment was carried out with ClustalW, while the phylogenetic tree was built using MEGA X. The classification was based on the *Arabidopsis* R2R3-MYB classification method. Phylogenetic trees were constructed using the adjacency method (NJ) and a Poisson model, with iTOL (https://itol.embl.de/tree/218210322139151769308754#, visit on 29 January 2026) subsequently being used for beautification. Using MEME tools version (5.3.3) (https://academic.oup.com/nar/article/43/W1/W39/2467905, visit on 25 January 2026), the PmMYB protein amino acid sequence was analyzed, and we set up 10 ordered sets. The lengths of the fragments ranged from 6 to 70 amino acids, with all other default parameters kept unchanged. Conserved motifs identified from the search were annotated with the SMART tool (http://smart.embl-heidelberg.de/, accessed 25 January 2026), and we used TBtools to conduct visual processing on the analyzed data.

### 4.3. Plant Materials and Processing

Propagation of eight-year-old Masson pine was carried out at Nanjing Forestry University (Nanjing, China). Four types of tissues, including lignified stems, semi-lignified stems, needles, and roots, were immediately frozen in liquid nitrogen and kept at −80 °C until RNA was extracted. Total RNA was extracted using the same RNA isolation kit as described above in accordance with the manufacturer’s instructions. Under suitable conditions (22 °C, a 16 h light period followed by an 8 h dark period), the tobacco seedlings (*Nicotiana benthamiana*) were planted in an aid-lit incubator. The seeds were treated with 10% sodium hypochlorite for 15 min for disinfection, followed by sowing in 1/2 MS medium. Following 2 days of simulated vernalization treatment (4 °C, darkness), the samples were placed in an incubator and incubated for 7 days. At 9 days post-sowing, seedlings were transplanted into a matrix mixed with 60% peat soil, 30% perlite, and 10% vermiculite.

### 4.4. Cloning of the Full-Length PmMYB289 Coding Sequence (CDS)

RNA extracted from the coniferous tissue of two-year-old Masson pine using the RNA isolation kit (CAT: RC-411-01) from Vazyme (Nanjing) Co., Ltd. (Nanjing, China) in accordance with the manufacturer’s instructions, and cDNA libraries obtained by reverse transcription using the rapid reverse transcription kit (11141ES60) from Yeasen Biotechnology (Shanghai) Co., Ltd. (Shanghai, China) Primers were designed for the full-length coding region sequence of the *PmMYB289* gene. The PCR product was inserted into the pBI121 vector, transformed into *Escherichia coli* DH5α, and subsequently sequenced. All primers employed in these experiments are presented in Supplementary Table S4.

### 4.5. Transcriptional Activation Assay of PmMYB289

The protein-coding sequence of *PmMYB289* was cloned into the pGBKT7 vector (Takara, Beijing, China; Table S4). The resulting recombinant plasmid was co-transformed with pGADK7 into the *Saccharomyces cerevisiae* AH109 strain via the LiAc/DNA/PEG method. Referring to the previous method, the transformed yeast cells were inoculated on SD/-T medium for the selection of positive transforms, then transferred to SD4-deficient medium without tryptophan (Trp), leucine (Leu), histidine (His), and adenine (Ade) for transcriptional activation assays. An empty pGBKT7 vector served as the negative control.

### 4.6. Subcellular Localization Assay

The full-length coding sequence of *PmMYB289* was inserted into the pBI121-GFP vector. The recombinant plasmid, as well as the empty control vector were transformed into *Agrobacterium tumefaciens* strain EHA105. After infiltration into tobacco leaves, which are 4 weeks old, these tobacco plants were cultured for 48–72 h (dark). The fluorescence signals of GFP were observed under a confocal laser scanning microscope. DAPI staining was used to indicate the position of the nucleus.

### 4.7. Agrobacterium-Mediated Tobacco Transformation

The binary vector plasmid pBI121 carrying the *PmMYB289* gene (Table S4), with *PmMYB289* controlled by the CaMV 35S constitutive promoter, was introduced into *Agrobacterium* strain EHA105. The leaf discs of tobacco were immersed and inoculated in a regenerated *Agrobacterium* infection suspension with OD600 = 0.6 and gently shaken at 200 revolutions per minute for 10 min. Subsequently, the leaf discs were dried, and co-culture was carried out for 2 days at 28 °C in the dark on MS medium (pH 5.8) containing 0.4 mg/L n-6-benzyladenine (6-BA), 0.1 mg/L 1-naphthylacetic acid (NAA), 0.01 mg/L thidiazuron (TDZ), 6 g/L agar, 30 g/L sucrose, and 200 mmol/L acetosyringone (AS). After that, the leaf discs were excised and transferred to fresh selection medium (pH 5.8) supplemented with the same basal concentrations of 6-BA, NAA, agar, sucrose, as well as 400 mg/L cefotaxime, and 50 mg/L kanamycin. Cultured at 25 °C under light/dark conditions for 16/8 h. In order to screen out possible transgenic explants. Then, the selected buds were first transferred onto a rooting medium of half-strength MS and then transplanted into the soil for supplementary experimentation. All genetically modified and wild-type plants were adapted and grew in greenhouses at Nanjing Forestry University under conditions of 18 to 23 °C;, 60% humidity, 18 h of light per day, and 6 h of darkness.

### 4.8. Semi-Quantitative Reverse Transcription Polymerase Chain Reaction and Quantitative Real-Time Polymerase Chain Reaction

Use the FastPure Plant Total RNA Isolation Kit (CAT: RC-411-01) from Vazyme (Nanjing) Co., Ltd. to extract total RNA from each sample in accordance with the manufacturer’s instructions. RNA concentration and purity were determined using the NanoDrop 2000 instrument (Thermo Fisher Scientific, Waltham, MA, USA). The first strand of cDNA was synthesized using the rapid reverse transcription kit (11141ES60) from Yeasen Biotechnology (Shanghai) Co., LTD. Primers designed for quantitative real-time reverse transcription PCR (qRT-PCR) using Primer 5.0 (Table S4). Detect the target sequence using SYBR Green reagent. Each PCR reaction system (10 μL) comprises 1 μL cDNA diluted 20-fold, 5 μL SYBR Green real-time PCR Master Mix, 0.4 μL 10 μM primer, and 3.2 μL ddH_2_O. The PCR protocol is composed of six stages: (1) 60 s at 95 °C (pre-denaturation); (2) 15 s at 95 °C; (3) 15 s at 60 °C; (4) 10 s at 72 °C, repeated 40 cycles for amplification; (5) 0.5 s at 95 °C; (6) 1 min at 60 °C (melting). Evaluate the quality of the PCR product based on the analysis of the melting curve. Use TUA (β-tubulin) as an internal reference. Three independent biological replicates and three technical replicates were performed separately. The relative expression levels of the target genes were calculated by employing the 2^−ΔΔCT^ method [44].

### 4.9. Data Analysis and Statistics

Each experiment was divided into three independent biological replicates. Significant differences at *p* < 0.05 (one-way ANOVA) are marked by distinct lowercase letters [Figure 4]. Statistical significance was determined by one-way ANOVA (* *p* < 0.05, ** *p* < 0.01) [Figure 7].

## 5. Conclusions

This study focuses on the R2R3-MYB transcription factor *PmMYB289* of Masson pine, aiming to clarify its role in secondary wall biosynthesis and provide a basis for improving pulpwood varieties. Genome-wide identification combined with phylogenetic analysis revealed a total of 317 R2R3-MYB genes in Masson pine, among which *PmMYB289* belongs to the P20 subgroup and is homologous to *Arabidopsis AtMYB52/54*. Tissue expression analysis showed it is highly expressed in old stems and needles, and yeast hybridization tests confirmed it has no transcriptional self-activation. Results from subcellular localization suggested that this protein is distributed in the nucleus. Overexpression of *PmMYB289* in tobacco significantly inhibited plant growth and most key secondary wall synthesis genes, though it did upregulate *NbPAL1* and had no effect on *NbCCoAOMT3/NbHCT*. Overall, PmMYB289 is a key transcription factor in the process of secondary wall biosynthesis. It can act as an inhibitory factor to suppress the formation of secondary walls in transgenic tobacco, which is of great significance for clarifying the mechanism of wood formation and cultivating high-quality Masson pine pulpwood varieties.

## Data Availability

The original contributions presented in this study are included in the article/Supplementary Material. Further inquiries can be directed to the corresponding author.

## Supplementary Materials

The following supporting information can be downloaded at: https://www.mdpi.com/article/10.3390/plants15081216/s1. Table S1: The characteristics and chromosomal localization of PmMYBs. Table S2: Protein sequences used for phylogenetic analysis of PmMYB gene family. Table S3: Classification correspondence between Masson pine and the *Arabidopsis* subfamily. Table S4: Primers used in this study. Figure S1: The gene structure of the R2R3-MYB family in Masson pine. Figure S2: Diagram showing the gene structure of the P20 subgroup of Masson pine.
