# Supplementary material for: Genome-Wide Identification of the R2R3-MYB Gene Family Members in Masson Pine and the Regulation of Secondary Cell Wall Formation and Lignin Biosynthesis by PmMYB289
Source: Plants (Basel). 2026 Apr 16;15(8):1216. doi: 10.3390/plants15081216 (PMC13120142; doi:10.3390/plants15081216)
Supplement: Supplementary file 1 [file plants-15-01216-s001.zip › Supplementary figures.pdf]

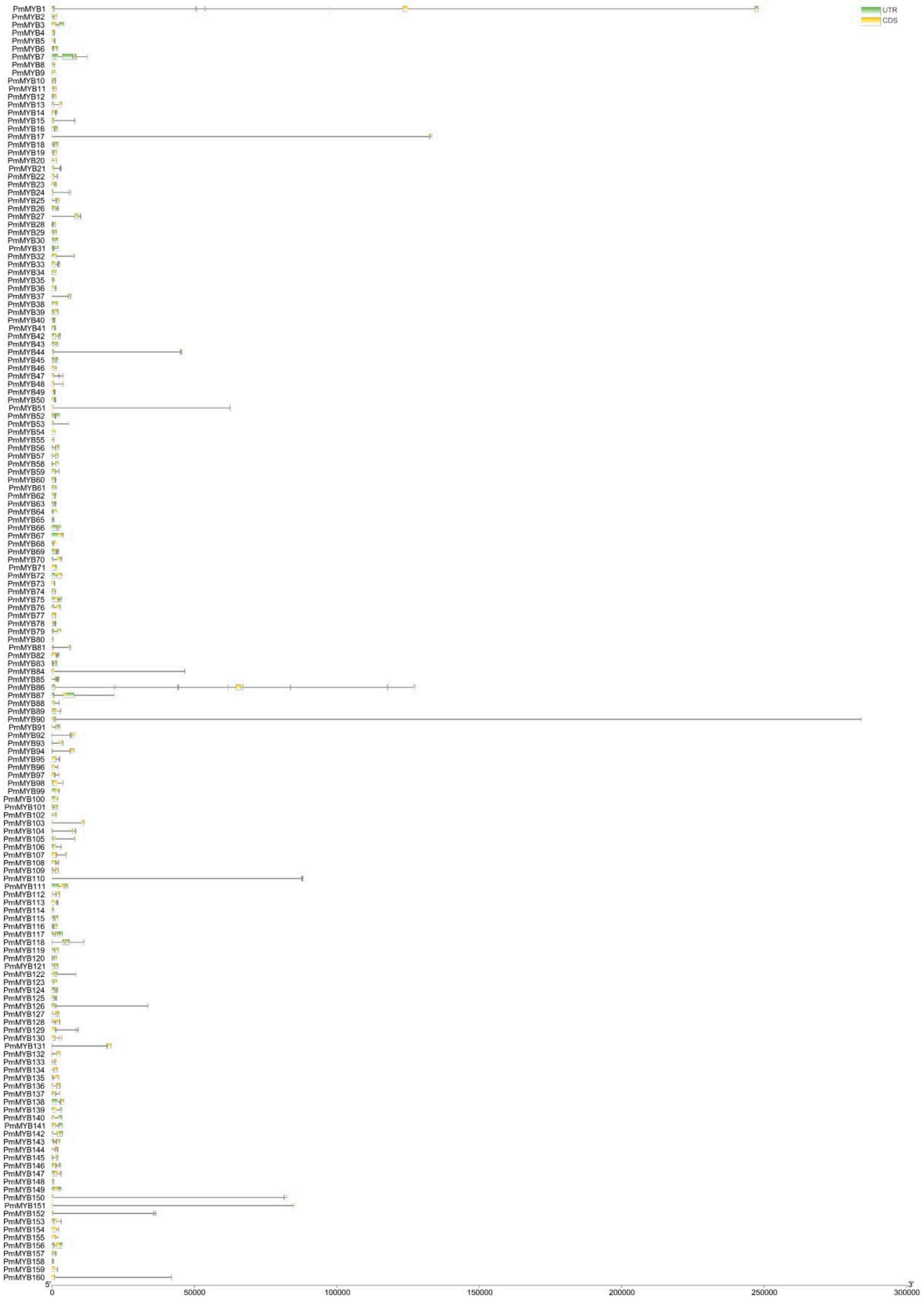

UTR
CDS
PmMYB1
PmMYB2
PmMYB3
PmMYB4
PmMYB5
PmMYB6
PmMYB7
PmMYB8
PmMYB9
PmMYB10
PmMYB11
PmMYB12
PmMYB13
PmMYB14
PmMYB15
PmMYB16
PmMYB17
PmMYB18
PmMYB19
PmMYB20
PmMYB21
PmMYB22
PmMYB23
PmMYB24
PmMYB25
PmMYB26
PmMYB27
PmMYB28
PmMYB29
PmMYB30
PmMYB31
PmMYB32
PmMYB33
PmMYB34
PmMYB35
PmMYB36
PmMYB37
PmMYB38
PmMYB39
PmMYB40
PmMYB41
PmMYB42
PmMYB43
PmMYB44
PmMYB45
PmMYB46
PmMYB47
PmMYB48
PmMYB49
PmMYB50
PmMYB51
PmMYB52
PmMYB53
PmMYB54
PmMYB55
PmMYB56
PmMYB57
PmMYB58
PmMYB59
PmMYB60
PmMYB61
PmMYB62
PmMYB63
PmMYB64
PmMYB65
PmMYB66
PmMYB67
PmMYB68
PmMYB69
PmMYB70
PmMYB71
PmMYB72
PmMYB73
PmMYB74
PmMYB75
PmMYB76
PmMYB77
PmMYB78
PmMYB79
PmMYB80
PmMYB81
PmMYB82
PmMYB83
PmMYB84
PmMYB85
PmMYB86
PmMYB87
PmMYB88
PmMYB89
PmMYB90
PmMYB91
PmMYB92
PmMYB93
PmMYB94
PmMYB95
PmMYB96
PmMYB97
PmMYB98
PmMYB99
PmMYB100
PmMYB101
PmMYB102
PmMYB103
PmMYB104
PmMYB105
PmMYB106
PmMYB107
PmMYB108
PmMYB109
PmMYB110
PmMYB111
PmMYB112
PmMYB113
PmMYB114
PmMYB115
PmMYB116
PmMYB117
PmMYB118
PmMYB119
PmMYB120
PmMYB121
PmMYB122
PmMYB123
PmMYB124
PmMYB125
PmMYB126
PmMYB127
PmMYB128
PmMYB129
PmMYB130
PmMYB131
PmMYB132
PmMYB133
PmMYB134
PmMYB135
PmMYB136
PmMYB137
PmMYB138
PmMYB139
PmMYB140
PmMYB141
PmMYB142
PmMYB143
PmMYB144
PmMYB145
PmMYB146
PmMYB147
PmMYB148
PmMYB149
PmMYB150
PmMYB151
PmMYB152
PmMYB153
PmMYB154
PmMYB155
PmMYB156
PmMYB157
PmMYB158
PmMYB159
PmMYB160
5'
3'
0
50000
100000
150000
200000
250000
300000

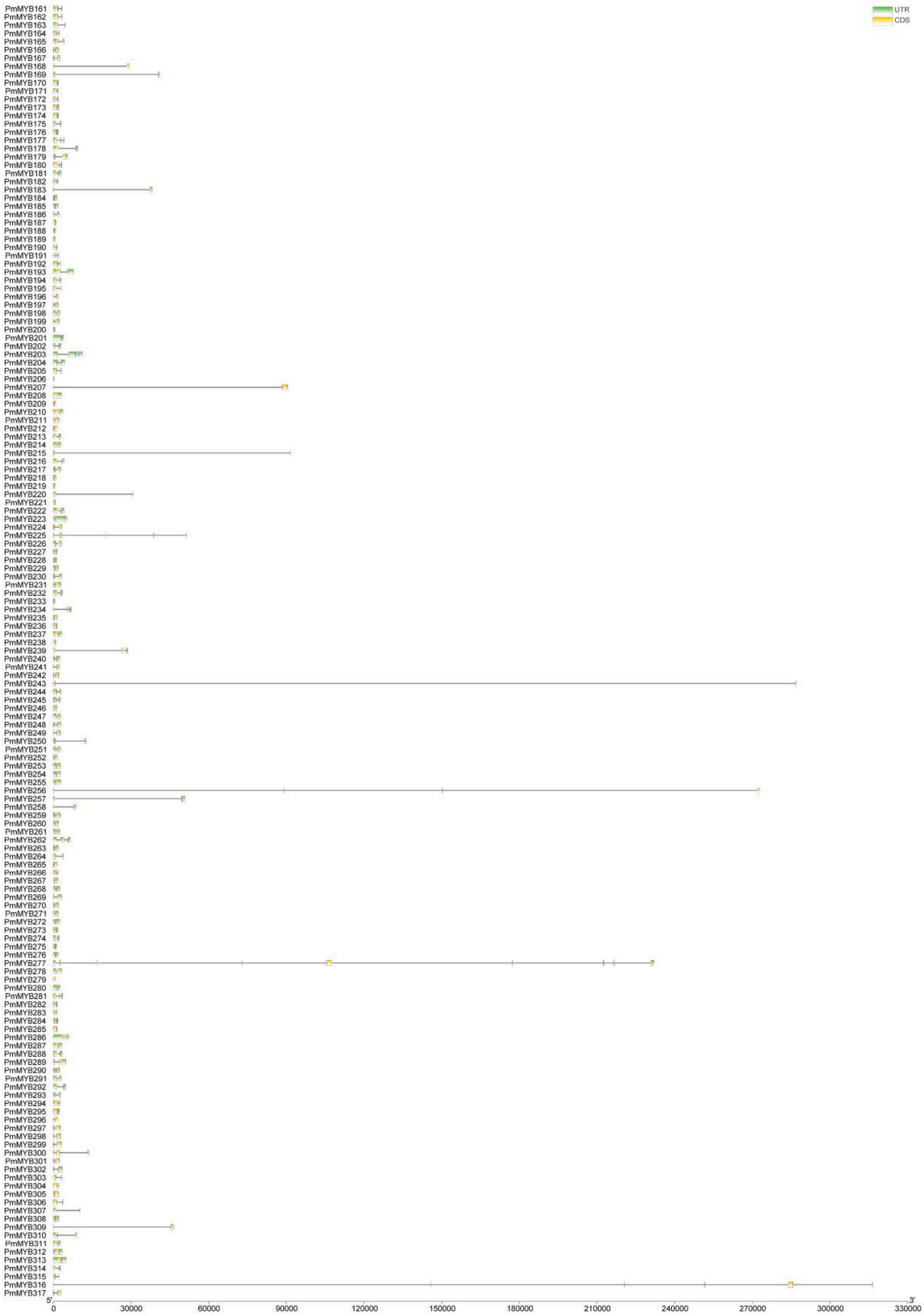

Figure S1. The gene structure of the R2R3-MYB family in Masson pine. Genes are arranged in the order of their names. The green blocks represent UTR, the yellow blocks represent CDS, and the black lines indicate introns.

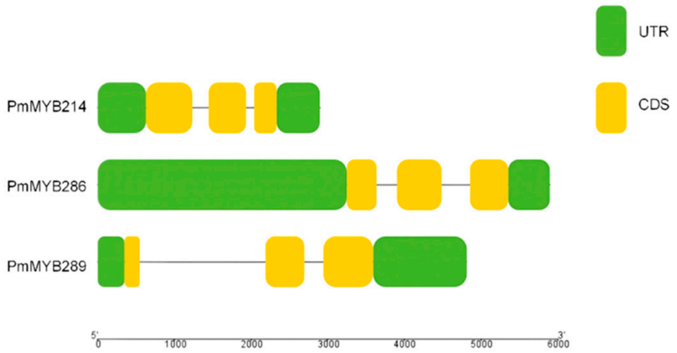

Figure S2. Diagram showing the gene structure of the P20 subgroup of Masson pine. All three genes contain three exons and three introns. Among them, *PmMYB214* has the shortest structure, with both UTR and introns being relatively short; *PmMYB286* has the longest total length, and its 5'UTR is also longer; *PmMYB289* has the longest 3'UTR and the longest first intron.
